# Supplementary material for: Frugoside Induces Mitochondria-Mediated Apoptotic Cell Death through Inhibition of Sulfiredoxin Expression in Melanoma Cells
Source: Cancers (Basel). 2019 Jun 19;11(6):854. doi: 10.3390/cancers11060854 (PMC6627655; doi:10.3390/cancers11060854)
Supplement: Supplementary file 1 [file cancers-11-00854-s001.pdf]

*Supplementary Materials*

# Frugoside Induces Mitochondria-Mediated Apoptotic Cell Death through Inhibition of Sulfiredoxin Expression in Melanoma Cells

**In-Sung Song, Yu Jeong Jeong, Ji Eun Kim, Jimin Shin and Sung-Wuk Jang**

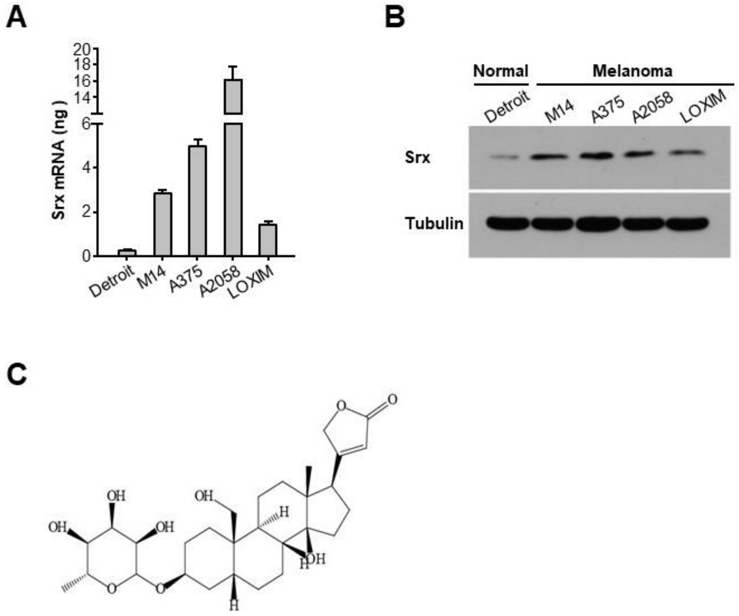


**Figure S1.** Srx expression in melanoma cells and the structure of frugoside, a compound validated for Srx inhibition. (**A**,**B**) Normal human skin cell line Detroit 551 and melanoma cell lines M14, A375, A2058, and LOX-IMVI were subjected to qPCR (**A**) with specific primers against the Srx gene and western blotting (**B**) with antibodies specific to Srx and tubulin. (**C**) The structure of frugoside is shown.

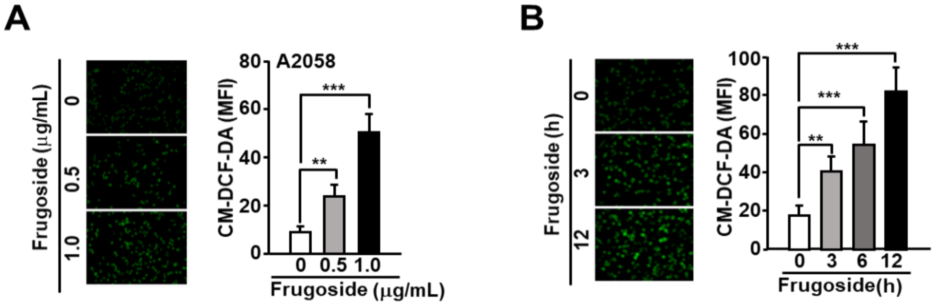


**Figure S2.** Frugoside induces ROS accumulation. (**A**,**B**) A2058 melanoma cells were treated with the indicated concentrations of frugoside for 12 h (**A**) or 0.5 μg/mL frugoside for the indicated time (**B**). After staining with 5 μM CM-H2DCFDA at 37 °C for 30 min, DCF fluorescence was measured by fluorescence microscopy. Intracellular ROS level was plotted as a function of incubation dose and time. Data represent mean ± SD. $p$ values were derived to assess statistical significance and are indicated as follows: ** $p < 0.01$; and *** $p < 0.001$. Original magfication, 100×.

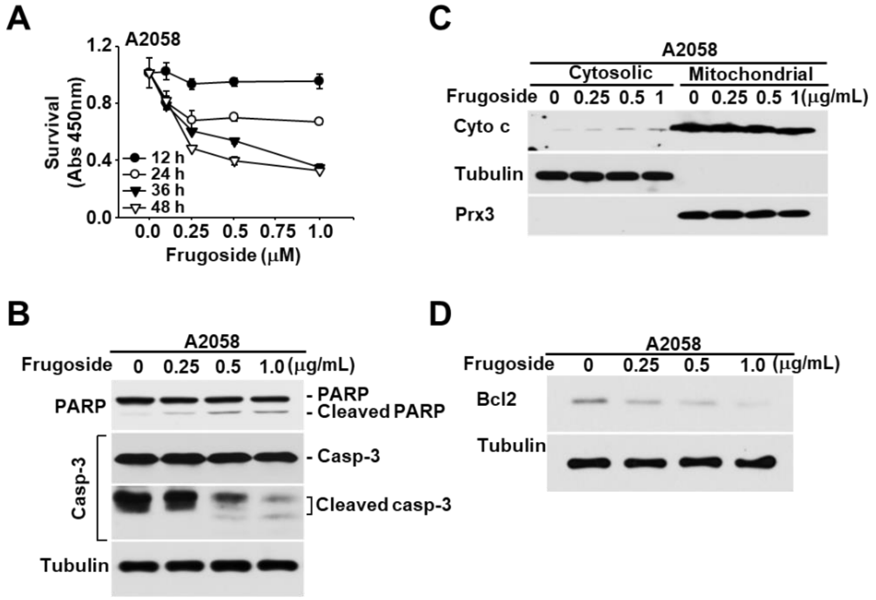


**Figure S3.** Frugoside induces mitochondria-mediated cell death in A2058 cells. (**A**) A2058 cells were treated with frugoside according to the indicated time and dose. The level of cytotoxicity was measured by the CCK-8 assay. (**B**). A2058 cells were treated with frugoside in a dose-dependent manner for 24 h and examined by western blotting using the indicated antibodies. (**C**) A2058 cells were treated with frugoside in a dose-dependent manner for 24 h and cell lysates were separated into cytosolic and mitochondrial fractions. Tubulin and Prx 3 were used as cytosolic and mitochondrial markers, respectively. (**D**) A2058 cells were treated with frugoside in a dose-dependent manner for 24 h and examined by western blotting using antibodies against Bcl2 and tubulin.

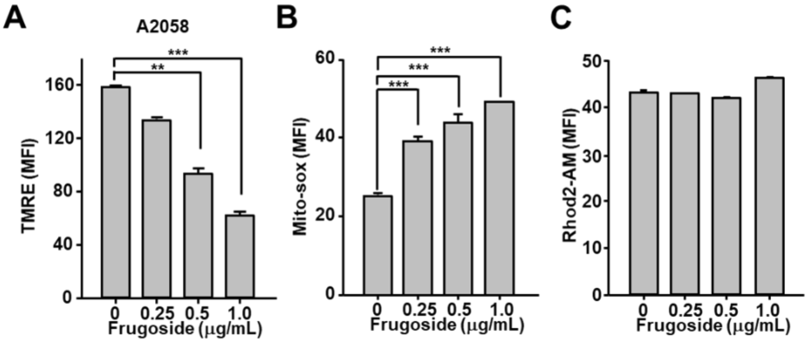


**Figure S4.** Frugoside results in mitochondrial- dysfunction via ROS overproduction. (**A**–**C**) Melanoma A2058 cells were treated with frugoside in dose-dependent manner for 24 h. The stimulated cells using a flow cytometer were analyzed for mitochondrial function, such as membrane potential (**A**), mitochondrial ROS (**B**), and calcium overload (**C**). Mitochondrial membrane potential was measured using a FACScanto II after TMRE staining in A2058 cells treated with frugoside according to the dose. Mitochondrial ROS was measured in frugoside-treated melanoma cells via Mito-Sox staining. Mitochondrial calcium level was measured using a Rhod2-AM fluorescence dye and quantified. Values are presented as mean ± SDs. $p$ values were derived to assess statistical significance and are indicated as follows: ** $p < 0.01$; and *** $p < 0.001$.

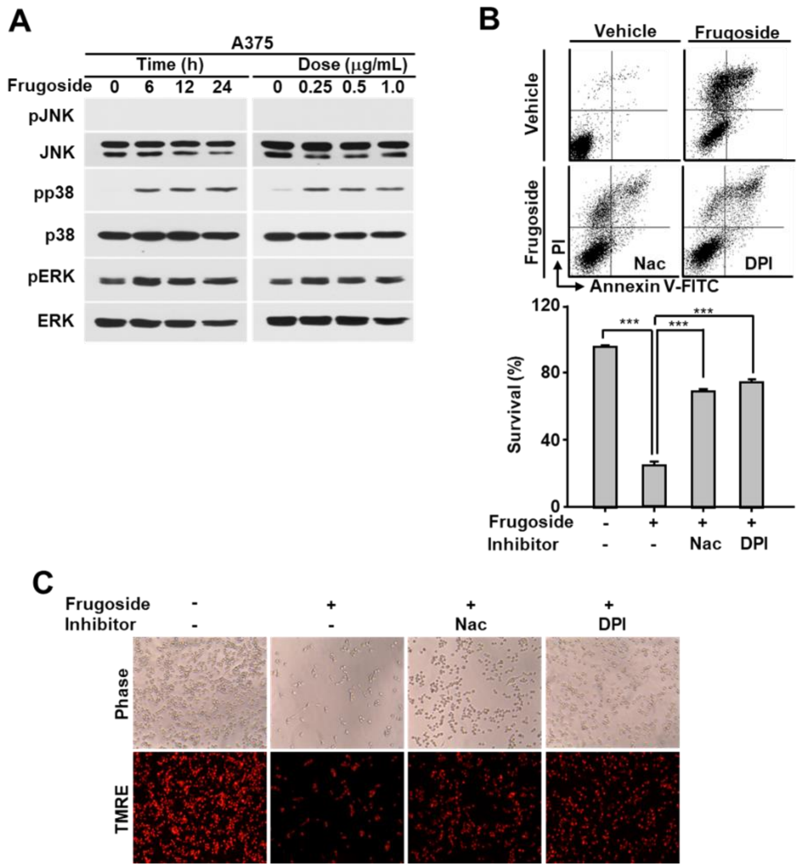


**Figure S5.** Accumulated ROS due to frugoside induces apoptotic cell death by sustained p38MAPK activation. (**A**) A375 cells were treated with frugoside in time- and dose-dependent manners. The cell lysates were subjected to western blotting with specific antibodies to phosphorylated MAPKs as indicated in the figure. The same membrane was re-probed with antibodies to the following MAPKs: JNK, p38MAPK, and ERK. (**B**) A375 cells were pretreated with 2 mM NAC or 10 μM DPI for 1 h, followed by 1 μg /mL frugoside for 24 h. The cells were subjected to FACS analysis after PI/Annexin V-FITC staining. (**C**) M14 cells were pretreated with 2 mM NAC or 10 μM DPI for 1 h, followed by 1 μg /mL frugoside for 24 h, and the cells were analyzed with a fluorescence microscope after TMRE staining. *p* values were derived to assess statistical significance and are indicated as follows: *** $p <$ 0.001. Original magfication, 100×.

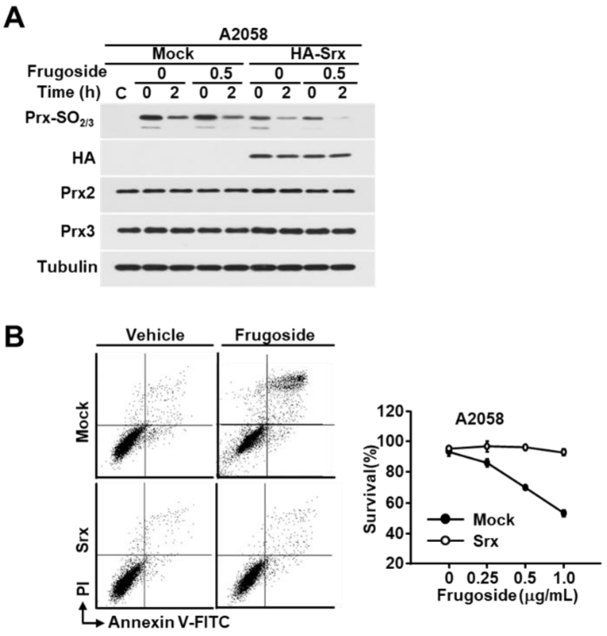


**Figure S6.** Srx overexpression rescues the ROS level and cell death in frugoside-treated cells. (**A**) A2058 cells were transfected with HA-Srx overexpressing plasmid or Mock, and then the cells were exposed to 200 μM $H_2O_2$ for 10 min in the presence of frugoside. After removal of $H_2O_2$, the cells were incubated for the indicated times in fresh medium. Sample C means control (no stimulation with $H_2O_2$). The cell lysates were subjected to western blotting analysis using antibodies specific for Prx-$SO_2$, Prx2, and Prx3. (**B**) A2058 cells were transfected with HA-Srx overexpressing plasmid or Mock, and the cells were treated with frugoside in a dose-dependent manner. Cell death was measured by FACS analysis after PI/Annexin V-FITC staining.

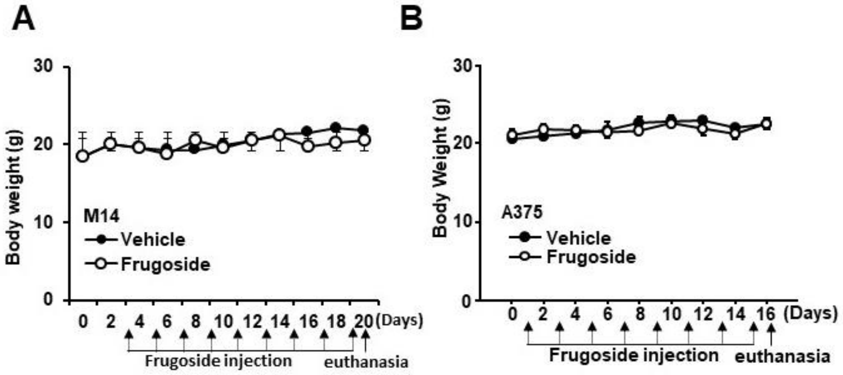


**Figure S7.** Frugoside inhibits tumorigenic ability in vivo. (**A**,**B**) To generate the xenograft model, 1 × $10^6$ M14 (**A**) or A375 (**B**) cells were subcutaneously injected into the flanks of 6-week-old female BALB/c nude mice. Frugoside (in 10% DMSO and 90% PBS) was intraperitoneally administered at doses of 100 μg/kg once every 2 d for 17 d. The change of body weight was monitored. All values represent mean ± SD, *n* = 5.

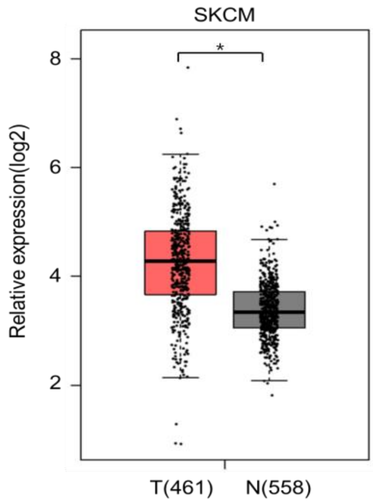


**Figure S8.** SRXN1 mRNA expression in normal and skin cutaneous melanoma (SKCM) based on TCGA and GTEx data in GEPIA. N, normal tissues; T, Tumor tissues. Number of samples indicated at the bottom, * $p < 0.05$, $p$-values between group were calculated using Welch's corrected $t$-test.

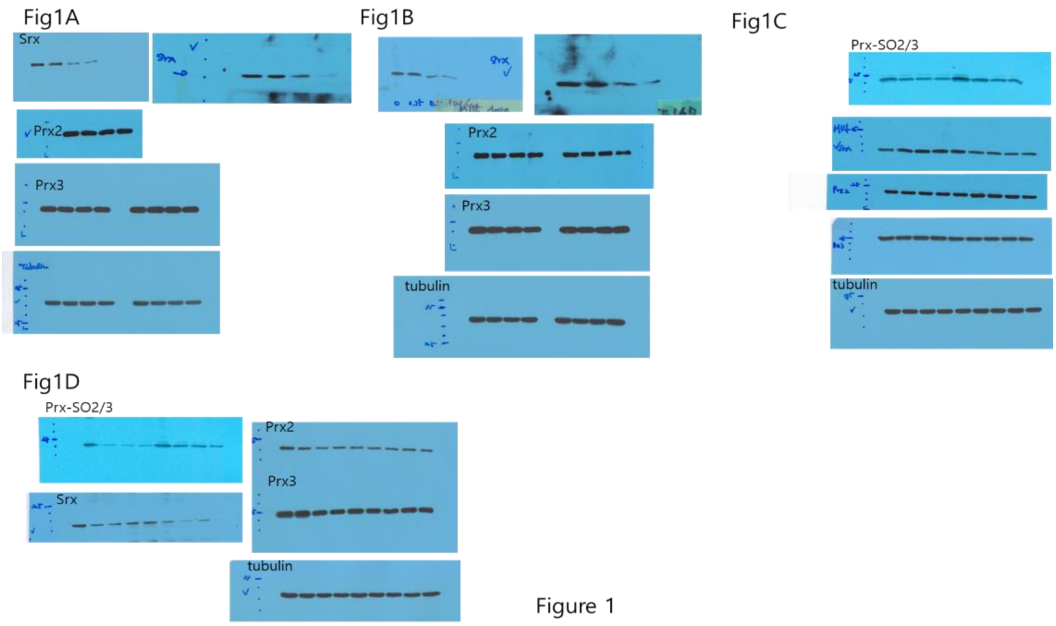


Figure 1

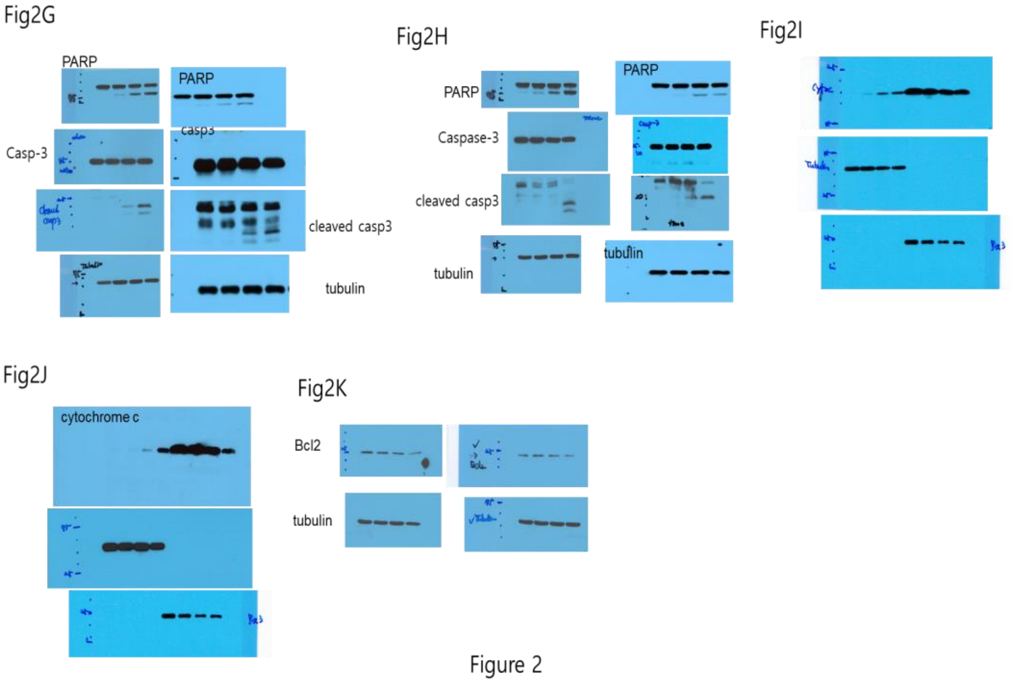


Figure 2

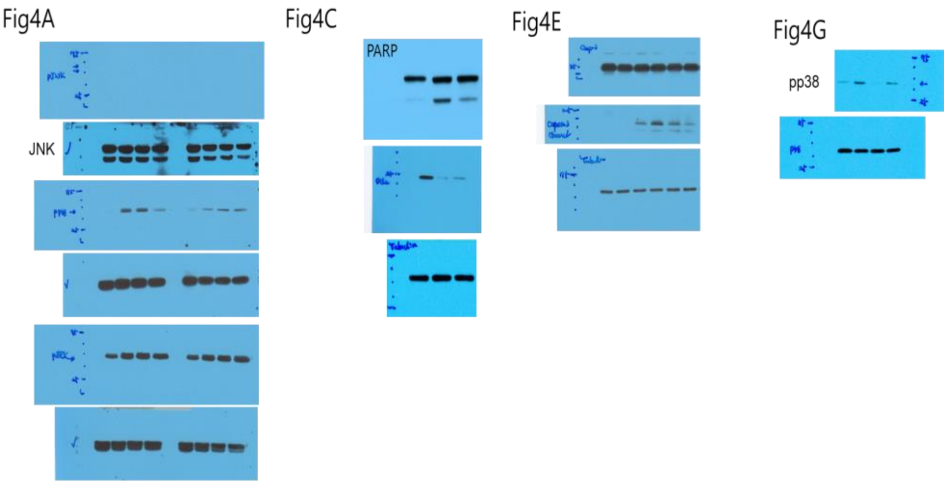


Figure 4

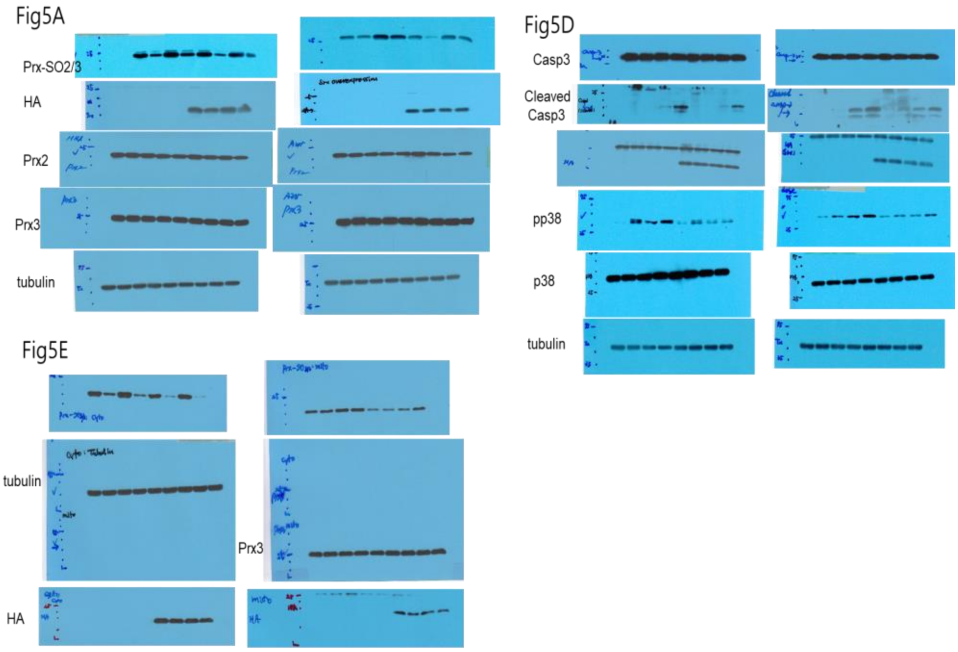


Figure 5

**Figure S9.** Uncropped images of western blot used in Figures.

© 2019 by the authors. Licensee MDPI, Basel, Switzerland. This article is an open access article distributed under the terms and conditions of the Creative Commons Attribution (CC BY) license (http://creativecommons.org/licenses/by/4.0/).
